# Coprescription of Chinese Herbal Medicine and Western Medications among Prostate Cancer Patients: A Population-Based Study in Taiwan

**DOI:** 10.1155/2012/147015

**Published:** 2011-07-18

**Authors:** Yi-Hsien Lin, Kuang-Kuo Chen, Jen-Hwey Chiu

**Affiliations:** ^1^School of Medicine, National Yang-Ming University, Taipei 112, Taiwan; ^2^Division of Radiotherapy, Cheng Hsin General Hospital, Taipei 112, Taiwan; ^3^Department of Surgery, Taipei Veterans General Hospital, Taipei 11217, Taiwan; ^4^Institute of Traditional Medicine, National Yang-Ming University, Taipei 112, Taiwan

## Abstract

Use of herbal medicine is popular among cancer patients. This study aimed to explore the coprescription of CHM and WM among prostate cancer patients in Taiwan. This cross-sectional retrospective study used a population-based database containing one million beneficiaries of National Health Insurance. Claims and prescriptions were analyzed. In 2007, 218 (22.4%) prostate cancer patients were CHM users. Among CHM users, 200 (91.7%) patients with 5618 (79.5%) CHM prescriptions were on coprescription of CHM and WM. A total of 484 types of CHM and 930 types of WM were used. The most commonly used CHMs on coprescription were Shu Jing Huo Xue Tang, Ma Zi Ren Wan, and Xue Fu Zhu Yu Tang. The most commonly used WMs on coprescription were magnesium oxide, amlodipine, and aspirin. The average number of prescriptions per user per year was 261.2 versus 151.7 in all (*P* < 0.001), 123.6 versus 76.9 in WM (*P* = 0.033), and 34.8 versus 5.1 in CHM (*P* < 0.001) for patients with and without coprescription, respectively. In conclusion, use of CHM among prostate cancer patients was popular in Taiwan. Most CHMs were used with WM concurrently. The potential drug-herb interactions should be investigated, especially for patients with more prescriptions.

## 1. Introduction

Complementary and alternative medicine (CAM) has become increasingly popular worldwide in the recent decades [1–3]. Herbal medicine is one of the most popular types of CAM. Previous studies showed 8.4–26.5% of prostate cancer patients using herbal remedies [4–8]. Chinese herbal medicine (CHM) has been used among the Chinese population for thousands of years and is gradually accepted in the West. Due to the possibility of drug-herb interactions, it is important to know which Chinese herbal medicine is most frequently used by prostate cancer patients. However, there is limited information on this issue.

National Health Insurance (NHI), which covers both Western and Chinese medicines, has been implemented in Taiwan since 1995. By 2010, over 99% of the 23 million residents are enrolled in the program. Beneficiaries are free to choose the types of medical services they prefer. NHI coverage of Chinese medicine services includes CHM, acupuncture, and traumatology manipulative therapies. 

The National Health Insurance Research Database (NHIRD) provides registration and claim datasets for research. In this study, we used NHIRD to explore the frequency and pattern of CHM use among prostate cancer patients. Coprescriptions of CHM and Western medications (WM) were also assessed.

## 2. Methods

### 2.1. Data Sources

This is a cross-sectional retrospective study using Longitudinal Health Insurance Database 2000 (LHID2000), which was obtained from NHIRD. LHID2000 contains all the original claim data of 1,000,000 individuals randomly sampled from the 23 million beneficiaries of the NHI. There is no significant difference in the distribution of age, gender and insured amount between the patients in the LHID2000 and the original NHIRD. Data in NHIRD that could be used to identify patients or care providers, including medical institutions and physicians, is scrambled before being sent to the National Health Research Institutes for database construction and is further encrypted before being released to each researcher. Since all the data had been deidentified, approval of institutional review board was exempt.

### 2.2. Study Samples

Under the NHI regulations, each claim for reimbursement is required to record up to three diagnosis codes in the format of International Classification of Diseases, Ninth Revision, Clinical modification (ICD-9-CM). Prostate cancer patients were identified from the file of ambulatory service of the year 2007 from LHID2000 with ICD-9-CM code 185. Claims and corresponding prescriptions of these prostate cancer patients in LHID2000 were then retrieved for analysis. Coprescription of WM and CHM was defined as the cases in which the two types of medication were prescribed within overlapped prescription duration.

### 2.3. Statistics

The database software ASIQ 12.5.7 (Sybase Inc, Dublin, Calif, USA) was used for data extraction and linking. The data were analyzed using SPSS for Windows Version 13.0 (SPSS Inc, Chicago, ILL, USA). The distribution and frequency of each category of variables were examined by Chi-square tests. A *P* value of less than 0.05 was considered statistically significant.

## 3. Results

A total of 972 prostate cancer patients were identified in the ambulatory service file of the year 2007 from LHID2000, with 42859 visits and 183108 prescriptions. Among them, 218 (22.4%) patients used CM, with 1361 visits (average 6.2 visits per user) and 7070 CHM prescriptions (average 5.2 prescriptions per visit). A total of 970 (99.8%) patients used Western medicine, with 32520 visits (33.5 visits per user) and 100736 WM prescriptions (average 3.1 prescriptions per visit).

### 3.1. Patient Demographics

The demographics are presented in Table 1. The median age was 75.4 in noncoprescription patients and 73.7 in coprescription patients. A higher proportion of coprescription patients were found at the age between 50 and 79 (*P* = 0.194).

With regard to income (insured payroll-related amounts), a higher portion of patients with monthly income of US$609–1218 was found in the group of coprescription (*P* = 0.18) (US$1 = NT$32.842).

In terms of insured regions, there was a higher proportion of coprescription patients registered in central Taiwan (*P* = 0.005).

As to insured unit, a higher proportion of coprescription patients were farmers and fishermen (*P* = 0.53).

### 3.2. Prescriptions

**Figure 1:**
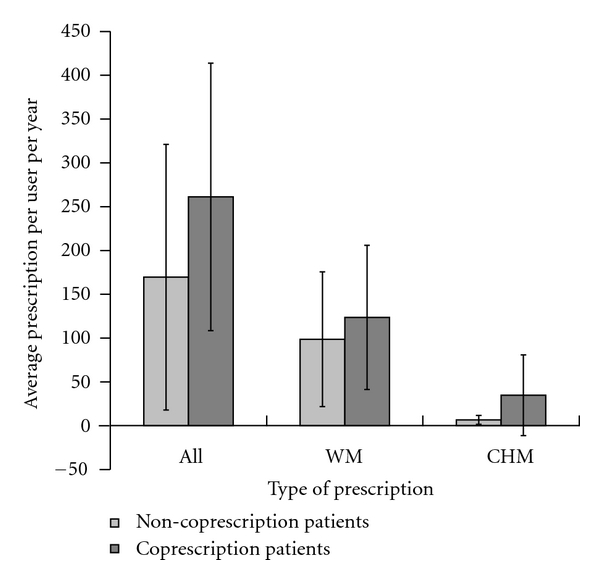
Average number of prescriptions per user per year of patients with and without coprescription. The average number of prescriptions per user per year was 261.2 versus 151.7 in all (*P* < 0.001), 123.6 versus 76.9 in WM (*P* = 0.033), and 34.8 versus 5.1 in CHM (*P* < 0.001) for patients with and without coprescription, respectively. Data were expressed as the mean ± SD. WM: Western medications; CHM: Chinese herbal medicine; All: WM and CHM.

The coprescription patients had more prescriptions than the noncoprescription patients (Figure 1). The average number of prescriptions per user per year was 261.2 versus 151.7 in all (*P* < 0.001), 123.6 versus 76.9 in WM (*P* = 0.033), and 34.8 versus 5.1 in CHM (*P* < 0.001) for patients with and without coprescription, respectively.

### 3.3. Prescriptions of Chinese Herbal Medicine

There were 7070 CHM prescriptions. A total of 484 types of CHM were used. The most frequently prescribed CHMs were Shu Jing Huo Xue Tang, Ma Zi Ren Wan, and Ji Sheng Shen Qi Wan (Table 2). Most CHM prescriptions were used for nonprostate-cancer diseases/conditions. There were 1510 (21.4%) prescriptions with diagnosis code of prostate cancer and 5560 (78.6%) prescriptions without diagnosis code of prostate cancer.

### 3.4. Prescriptions of Western Medications

The WM prescriptions amounted to 100736. A total of 930 types of WMs were used. The most frequently prescribed WM were acetaminophen, aspirin, and tamsulosin (Table 2). Most WM prescriptions were used for nonprostate-cancer diseases/conditions. There were 26699 (26.5%) prescriptions with diagnosis code of prostate cancer and 74037 (73.5%) prescriptions without diagnosis code of prostate cancer.

### 3.5. Coprescription of CHM and WM

Among CHM users, 200 (91.7%) patients had coprescriptions of CHM and WM. A total of 5618 (79.5%) CHM prescriptions were used with WM concurrently. In coprescribed prescriptions, 468 types of CHM and 506 types of WM were found. The most frequently used CHMs in coprescription were Shu Jing Huo Xue Tang, Ma Zi Ren Wan, and Xue Fu Zhu Yu Tang. The most frequently used WMs in coprescription were magnesium oxide, amlodipine, and aspirin (Table 3).

The prescriptions of CHM and WM for cancer and noncancer diseases were quite different. For visits with diagnosis code of prostate cancer, the most common types of CHM for coprescription were Bai Mao Gen, Ma Zi Ren Wan, and Da Huang and the most common types of WM for coprescription were cyproterone acetate, tamsulosin, and magnesium oxide (Table 4).

Among the 237 prostate cancer patients who used cyproterone acetate, 29 (12.2%) patients used CHM and cyproterone acetate concurrently, with 665 (41.4%) CHM prescriptions.

## 4. Discussion

Use of herbal medicine among prostate cancer patients is popular. Yet there is little information readily available on what CHM products are used by prostate cancer patients. This is the first population-based study to provide the empirical evidence of coprescription of CHM and WM among prostate cancer patients. With over 90% coverage rate of NHI, the NHIRD is representative of the whole population in Taiwan. The complete claims of prescriptions in different medical institutes of the sample patients were obtained for analysis. Our results demonstrated that 22.4% prostate cancer patients used CHM. A total of 484 types of CHM were used. Most prescribed CHM have seldom been reported for prostate cancer in literature even though they were popularly used in this study. Most CHM prescriptions (79.5%) were used with WM concurrently. The potential drug-herb interactions should be explored. This study provided relevant information for the subsequent basic and clinical studies.

In Taiwan, only licensed Chinese medicine physicians are qualified for reimbursement of CM services under NHI. Services of Chinese medicine include CHM, acupuncture, and traumatology manipulative therapies. Reimbursement for CHM is limited to concentrated Chinese medicines registered as “only for dispensing a prescription” and “prescription only used by physicians (Chinese medicine).” Compounding concentrated Chinese medicines shall be listed in “Standard formula of clinically common used Chinese medicine” edited by central health authority. All NHI-covered CHMs should be manufactured by approved good manufacturing practice (GMP) Chinese medicine companies. Therefore, the quality control and quality assurance of CHM used in NHI are under surveillance by the central health authority in Taiwan. According to the regulations, Chinese medicine physicians are certified to prescribe CHM but not WM. Western doctors are certified to prescribe WM but not CHM.

The prevalence of CHM is much higher than that in our previous cross-sectional study [9] and is consistent with our previous long-term trends studies [10]. Our previous cross-sectional study only accounted CM visits with diagnosis code of prostate cancer (ICD-9-CM code 185) and found 2.6% prostate cancer patients used CM services. Our previous long-term trends study explored CM use among prostate cancer patients under NHI from 1996 to 2008 and found that the prevalence of CM use in each cross-sectional year increased slightly from 24.9% to 25.6%. Most CM visits (92.7%) were noncancer-specific. The most frequently used CM therapy was CHM (72.8% to 78.8%). In this study, all CM visits of prostate cancer patients were selected including claims with and without diagnosis code of prostate cancer. This study found only 21.4% prescriptions with diagnosis code of prostate cancer. Most prostate cancer patients used CHM for nonprostate-cancer diseases/conditions. Since most CHM prescriptions had been given without diagnosis code of prostate cancer, it is very likely that many Chinese medical physicians were not aware of the diagnosis of prostate cancer of these patients.

This study found the patients with coprescription had more prescriptions than the patients without coprescription. Prostate cancer is particularly common among elderly men. Given the higher prevalence of multiple health conditions in elderly, inappropriate medication use and adverse drug events (ADEs) are substantial problems in this population [11, 12]. The number of medications consumed is the main risk factor of inappropriate medication use and ADE [11, 12]. The relationship between number of prescriptions and ADE risk is strong [13, 14]. Our study found much higher average prescriptions in the study population than previous study. The risk of ADE needs further study.

A total of 484 types of CHM were used in this study. The most frequently used CHMs were Shu Jing Huo Xue Tang, Ma Zi Ren Wan, and Ji Sheng Shen Qi Wan. According to the concept of Chinese medicine, Shu Jing Huo Xue Tang is used for serous arthritis of the knees, lower back pain, sciatica, numbness in the lower limbs, and edema of the legs. This is consistent with acetaminophen and aspirin being the most commonly used WM. Ma Zi Ren Wan is a formula and is used to lubricate the intestines and consequently promotes bowel movement. Ji Sheng Shen Qi Wan is used for kidney Yang deficiency, heaviness at lumbar, foot edema, and difficulty in urination. The concept of prescribing CHM is different from that of prescribing WM. According to Chinese medicinal principles, the development of cancer is viewed as the result of an imbalance of the whole body-mind network. The kidney is the organ that maintains balance of the entire urological system. Therefore, Chinese medicine physicians will prescribe herbs or formulas to restore the balance of *yin* and *yang* in the kidneys rather than to treat cancer directly. 

There is no consensus on the standard CHM regimens for prostate cancer currently. This study revealed the complexity of CHM use among prostate cancer patients as the most common CHM represents only 111 out of 7070 in total (1.6%). This reflected the multiple symptoms of prostate cancer patients presented to CM physicians and the disagreement in prescription policy among CM physicians. The theory of CM is very distinct from that of WM. CM physicians treat symptoms rather than diseases. For prostate cancer patients who experienced different symptoms, CM physicians would prescribe different CHM regimens. Besides, one CHM prescription usually combines several single herbs and formulas to balance the toxic effects. In the choice of herbs and formulas, there are also many alternative combinations with similar effects. Therefore, CHM use for prostate cancer is complex.

The top ten most common CMH prescriptions among prostate cancer patients contained 43 types of single herbs. Some herbs were more commonly used than others. Since these herbs were so popular, they might help to improve quality of life among prostate cancer patients. Their effects in prostate cancer deserve further investigation. On the other hand, the risk of drug-herb interactions of these herbs would be higher. Licorice (*Radix Glycyrrhizae*) and Niu Xi (*Radix Achyranthis Bidentatae)* were used in four of the top ten CMH prescriptions. Licorice is a very popular CHM and commonly used in herbal formulae to “harmonize” the other ingredients. Licorice was used for the treatment of peptic ulcer, constipation, cough, and other diseases. Clinical and experimental studies suggested that it has several other useful pharmacological properties such as antiinflammatory, antiviral, antimicrobial, antioxidative, anticancer activities, and immunomodulatory, hepatoprotective, and cardioprotective effects [15, 16]. Licorice was reported to interact with angiotensin-converting enzyme (ACE) inhibitors, diuretics, aspirin, digoxin, corticosteroids, insulin, and laxatives [17–19]. Niu Xi is used for expeling blood stasis, nourishing the liver and kidney, and strengthening the bones and muscles. Niu Xi was reported to have antiinflammatory, analgesic and antihypertensive effects [20]. The use of Niu Xi along with diuretics and antihypertensive drugs may result in additive effects.

Most CHM products are used for centuries in Chinese population and are generally safe if CHM products are used under the traditional rules. Our study found that most CHM prescriptions (79.5%) were used with WM concurrently. Appropriate integration of CHM and WM may have synergistic effects; in consequence, treatment outcome is enhanced and side effects are suppressed. However, the potential and significance of interactions between most CHM and WM are not widely recognized. Recent studies reported that some herbs had the potential to cause drug-herb interactions with anticancer drugs or other medications [21–25]. Among coprescribed prescriptions, 468 types of CHM and 506 types of WM were used. Many CHM products were herbal formulas, containing a variety of herbs. The most frequently used CHMs in coprescription were Shu Jing Huo Xue Tang, Ma Zi Ren Wan, and Xue Fu Zhu Yu Tang. The most frequently used WMs in coprescription were magnesium oxide, amlodipine, and aspirin. Adverse interactions between herbs and analgesic drugs were reported, such as acetaminophen and aspirin [26]. Concurrent use of Ginseng (*Panax Ginseng*) and amlodipine has the potential to cause adverse interactions [27]. Ginseng may also cause additive blood glucose-lowering effects when used in combination with antidiabetic drugs and thus increase the risk of hypoglycemia [19]. Dong Quai (*Radix Angelicae Sinensis*), Dan Shen (*Radix Salviae Miltiorrhizae*), Chinese wolfberry (*Fructus Lycii*) and Ginseng were reported to have interactions with warfarin [28]. Ginger (*Rhizoma Zingiberis Recens*) has the potential to interact pharmacologically with aspirin and coumarin derivatives [28]. Ginkgo was reported to have possible anticoagulant effects, which would interfere with chemotherapy and radiation therapy [29]. Fu Ling (*Poria*) was reported to interact with diuretics [19, 30].

The prescriptions of CHM and WM for cancer and noncancer diseases were quite different. For visits with diagnosis code of prostate cancer, the most frequently used CHM products in coprescription were Bai Mao Gen (*Rhizoma Imperatae Cylindricae*), Ma Zi Ren Wan, and Da Huang (*Radix et Rhizoma Rhei*) and the most frequently coprescribed WM were cyproterone acetate, tamsulosin, and magnesium oxide. Cyproterone acetate is oral nonsteroidal antiandrogen and used in the treatment of prostate cancer. Among 237 prostate cancer patients who used cyproterone acetate, 29 (12.2%) patients used CHM and cyproterone acetate concurrently, with 665 (41.4%) CHM prescriptions. Interactions of herbs with antiestrogenic drugs have been reported [31, 32]. Licorice and Dong Quai (*Radix Angelicae Sinensis*) have the potential of interactions with estrogen [19]. However, there is little information on the interactions between herbs and antiandrogen drugs. Because antiandrogen drugs had been used for anticancer purpose for years, the potential interactions of antiandrogen drugs with herbs are important problems. Further investigation on this issue is necessary.

This study found coprescription patients tended to be at age 50–79 (no significant difference), with a middle level of monthly income of US$609–1218 (no significant difference), living in central Taiwan (statistical significance), and belonging to the career category of farmers or fishermen (no significant difference). The manpower of CM physicians was geographical maldistributed in Taiwan. There are more CM physicians and institutes per person in central Taiwan than in other regions [33]. A past study also showed that “usable resources” was an important factor which influenced the CM purchasing behavior [34]. Because most CHM prescriptions were used with WM concurrently, patients in central Taiwan showed a higher rate of coprescription.

The strength of this study is that the data is representative and reliable. Since the NHI covered 99% of Taiwan's 23 million residents and over 90% of the medical institutes, the NHIRD is representative of the general population. NHIRD provides clear and reliable records and avoids recall bias in answering survey questions on past utilization. Besides, reimbursements for Chinese medicine were restricted to licensed Chinese medicine physicians who practiced at hospitals or clinics. Our study assessed the utilization of legally recognized Chinese medicine, the most important part of CAM in Taiwan's health system. Our previous study showed prostate cancer patients visited Western medicine physicians at hospitals and visited Chinese medicine physicians at clinics. NHIRD provides all the claim dataset for study and is a good tool to investigate concurrent use of medications prescribed by different medical institutes.

There are several limitations in this study. Because NHI only covered ambulatory services for Chinese medicine, this study only accounted claims of ambulatory services but not inpatients services. Medications at admission and postdischarge medications were not accounted. Besides, insurance-covered CHM is restricted to concentrated Chinese herbal medicine. Raw and over-the-counter herbal products were not covered by NHI and therefore not accounted in this study. CHM is very popular in Taiwan. Liu's study surveyed 64 cancer patients and found that only about one-thirds of CHM products were obtained from Chinese medical clinics (with licensed practitioner) [35]. It is estimated that the same or double amount of CHM was used by the patients themselves. The prevalence of concurrent use of CHM and WM was expected to be higher. Finally, the NHIRD is established primarily for administrative purposes. Clinical characteristics, including staging and biochemical data were not available. The outcome and side effects of CHM use were not assessed in this study.

In conclusion, use of Chinese herbal medicine among prostate cancer patients was popular in Taiwan. Most Chinese herbal medicines were used with Western medications concurrently. The potential drug-herb interactions should be investigated, especially for patients with more prescriptions.

## Figures and Tables

**Table 1:** Patient demographics.

Characteristics	Patients without coprescription		Patients with coprescription		*P* value
	Total	%	Total	%	
No. of patients	772		200		
Age					0.194
Median (years)	75.8		73.7		
<50	17	2.2	5	2.5	
50's	59	7.6	21	10.5	
60's	155	20.1	46	23.0	
70's	296	38.3	81	40.5	
>80	245	31.7	47	23.5	
Insured amount (US$/month)					0.18
0	220	28.5	55	27.5	
1–608	286	37.0	67	33.5	
609–1218	194	25.1	64	32.0	
>1218	72	9.3	13	6.5	
Missing data	0	0.0	1	0.5	
Insured region					0.005
Northern Taiwan	472	61.1	99	49.5	
Central Taiwan	108	14.0	46	23.0	
Southern Taiwan	175	22.7	51	25.5	
Eastern Taiwan & Offshore islands	17	2.2	3	1.5	
Missing data	0	0.0	1	0.5	
Insured unit		0.0			
Employees of government, school, enterprises or institutions	88	11.4	21	10.5	0.53
Members of occupational unions, alien seamen	25	3.2	4	2.0	
Farmers, Fishermen	171	22.2	54	27.0	
Low-income households	3	0.4	2	1.0	
Veterans, other regional population^a^	265	34.3	63	31.5	
Dependent^b^	220	28.5	55	27.5	
Missing data	0	0.0	1	0.5	

^a^All family members are unemployed and not low-income households.

^b^People are unemployed or under 20 years of age and have insured family member.

**Table 2:** Top ten most common prescriptions among prostate cancer patients.

Rank	Western medications (*n* = 100736)			Chinese herbal medications (*n* = 7070)		
	Products (compound)	*n*	%	Products (Chinese name)	*n*	%
1	Acetaminophen	3467	3.4	Shu Jing Huo Xue Tang	111	1.6
2	Aspirin	2366	2.3	Ma Zi Ren Wan	99	1.4
3	Tamsulosin	1928	1.9	Ji Sheng Shen Qi Wan	89	1.3
4	Magnesium oxide	1779	1.8	Yan Hu Suo	75	1.1
5	Cyproterone acetate	1610	1.6	Xue Fu Zhu Yu Tang	73	1.0
6	Amlodipine	1600	1.6	Ping Wei Shen	70	1.0
7	Doxazosin	1258	1.2	Da Huang	68	1.0
8	Sennoside	1242	1.2	Du Huo Ji Sheng Tang	67	0.9
9	Metformin	1230	1.2	Wu Wei Zi	66	0.9
				Zhi Bai Di Huang Wan	66	0.9
10	Sodium chloride	1153	1.1	Jie Geng	64	0.9

**Table 3:** Top ten most common CHMs and WMs used in coprescription among prostate cancer patients.

Rank	Western medications (*n* = 5369)			Chinese herbal medications (*n* = 5617)		
	Products (compound)	*n*	%	Products (Chinese name)	*n*	%
1	Magnesium oxide	138	2.6	Shu Jing Huo Xue Tang	102	1.8
2	Amlodipine	136	2.5	Ma Zi Ren Wan	97	1.7
3	Aspirin	120	2.2	Xue Fu Zhu Yu Tang	66	1.2
4	Acetaminophen	111	2.1	Zhi Bai Di Huang Wan	63	1.1
5	Tamsulosin	98	1.8	Da Huang	62	1.1
				Du Huo Ji Sheng Tang	62	1.1
6	Metformin	75	1.4	Jie Geng	59	1.1
7	Cyproterone acetate	71	1.3	Ping Wei Shen	58	1.0
8	Ambroxol	65	1.2	Yan Hu Suo	57	1.0
	Terazosin	65	1.2			
9	Sennoside	61	1.1	Wu Wei Zi	53	0.9
				Ji Sheng Shen Qi Wan	53	0.9
10	Dipyridamole	59	1.1	Niu Xi	50	0.9

**Table 4:** Top ten most common Western medications and Chinese herbal medications used in coprescription of claims with diagnosis code of prostate cancer.

Rank	Western medications (*n* = 1532)			Chinese herbal medications (*n* = 1146)			
	Products (compound)	*n*	%	Products (Chinese name)	Ingredients (Latin name)	*n*	%
1	Cyproterone acetate	70	4.6	Bai Mao Gen	*Rhizoma Imperatae Cylindricae*	40	3.5

2	Tamsulosin	63	4.1	Ma Zi Ren Wan	*Fructus Cannabis, Radix Paeoniae, Fructus Aurantii Immaturus, Radix et Rhizoma Rhei, Cortex Magnoliae Officinalis, Semen Armeniaccae Amarum*	35	3.1

3	Magnesium oxide	60	3.9	Da Huang	*Radix et Rhizoma Rhei*	31	2.7
				Sheng Di Huang	*Radix Rehmanniae*	31	2.7

4	Imipramine	50	3.3	Zuo Gui Wan	*Radix Rehmanniae, Glutinosae Conquitae, Fructus Corni Officinalis, Fructus Lycii, Colla Cornus Cervi, Semen Cuscutae Chinensis, Radix Dioscoreae Oppositae, Colla Carapacis et Plastri Testudinis, Radix Achyranthis Bidentatae*	29	2.5

5	Terazosin	49	3.2	Niu Xi	*Radix Achyranthis Bidentatae*	28	2.4

6	Leuprolide acetate	43	2.8	Che Qian Zi	*Semen Plantaginis*	27	2.4

7	Tolterodine L-tartrate	40	2.6	Yu Mi Xu	*Stigma Maydis*	26	2.3
				Zhu Ru	*Caulis Bambusae in Taeniis*	26	2.3

8	Doxazosin	34	2.2	Gui Zhi Fu Ling Wan	*Ramulus Cinnamomi, Sclerotium Poriae Cocos, Cortex Moutan Radicis, Semen Persicae, Radix Paeoniae Rubra*	26	2.3

9	Sennoside	30	2.0	Gui Lu Er Xian Jiao	*Colla Carapacis et Plastri Testudinis, Colla Cornus Cervi, Fructus Lycii, Radix Ginseng*	23	2.0

10	Alfuzosin	29	1.9	Shu Jing Huo Xue Tang	*Radix Glycyrrhizae, Radix Angelicae Sinensis, Radix Paeoniae Alba, Radix Rehmanniae, Rhizoma Atractylodis, Radix Cyathulae, Pericarpium Citri Reticulatae, Semen Persicae, Radix et Rhizoma Clematidis, Rhizoma Chuanxiong, Radix Stephaniae Tetrandrae, Rhizoma et Radix Notopterygii, Radix Saposhnikoviae, Radix Angelicae Dahuricae, Gelatinum Plastri Testudinis, Poria, Rhizoma Zingiberis Recens*	22	1.9
